# Night shift work exposure profile and obesity: Baseline results from a Chinese night shift worker cohort

**DOI:** 10.1371/journal.pone.0196989

**Published:** 2018-05-15

**Authors:** Miaomiao Sun, Wenting Feng, Feng Wang, Liuzhuo Zhang, Zijun Wu, Zhimin Li, Bo Zhang, Yonghua He, Shaohua Xie, Mengjie Li, Joan P. C. Fok, Gary Tse, Martin C. S. Wong, Jin-ling Tang, Samuel Y. S. Wong, Jelle Vlaanderen, Greg Evans, Roel Vermeulen, Lap Ah Tse

**Affiliations:** 1 JC School of Public Health and Primary Care, the Chinese University of Hong Kong, Hong Kong SAR, China; 2 Shenzhen Prevention and Treatment Center for Occupational Diseases, Shenzhen, China; 3 Shenzhen Municipal Key Laboratory for Health Risk Analysis, Shenzhen Research Institute of the Chinese University of Hong Kong, Shenzhen, China; 4 School of Public Health, Sun Yat-sen University, Guangzhou, China; 5 Department of Molecular Medicine, Karolinska Institutet, Karolinska University Hospital, Stockholm, Sweden; 6 Occupational Medicine Care Service, New Territories East Cluster, Hospital Authority, Hong Kong SAR, China; 7 Department of Medicine and Therapeutics, Faculty of Medicine, the Chinese University of Hong Kong, Hong Kong SAR, China; 8 Institute for Risk Assessment Sciences, Utrecht University, Utrecht, Netherland; 9 Occupational and Environmental Health Division, Dalla Lana School of Public Health, University of Toronto, Toronto, Canada; University of Lübeck, GERMANY

## Abstract

**Aims:**

This study aimed to evaluate the associations between types of night shift work and different indices of obesity using the baseline information from a prospective cohort study of night shift workers in China.

**Methods:**

A total of 3,871 workers from five companies were recruited from the baseline survey. A structured self-administered questionnaire was employed to collect the participants’ demographic information, lifetime working history, and lifestyle habits. Participants were grouped into rotating, permanent and irregular night shift work groups. Anthropometric parameters were assessed by healthcare professionals. Multiple logistic regression models were used to evaluate the associations between night shift work and different indices of obesity.

**Results:**

Night shift workers had increased risk of overweight and obesity, and odds ratios (ORs) were 1.17 (95% CI, 0.97–1.41) and 1.27 (95% CI, 0.74–2.18), respectively. Abdominal obesity had a significant but marginal association with night shift work (OR = 1.20, 95% CI, 1.01–1.43). A positive gradient between the number of years of night shift work and overweight or abdominal obesity was observed. Permanent night shift work showed the highest odds of being overweight (OR = 3.94, 95% CI, 1.40–11.03) and having increased abdominal obesity (OR = 3.34, 95% CI, 1.19–9.37). Irregular night shift work was also significantly associated with overweight (OR = 1.56, 95% CI, 1.13–2.14), but its association with abdominal obesity was borderline (OR = 1.26, 95% CI, 0.94–1.69). By contrast, the association between rotating night shift work and these parameters was not significant.

**Conclusion:**

Permanent and irregular night shift work were more likely to be associated with overweight or abdominal obesity than rotating night shift work. These associations need to be verified in prospective cohort studies.

## Introduction

Prolonged exposure to shift work, particularly to irregular night shifts, has been associated with higher risks of obesity, metabolic syndrome, cardiovascular diseases, and breast cancer [1, 2]. In China, the prevalence of overweight and obesity has risen rapidly in the last 20 years, collectively accounting for overweight or obesity in 1 in 3 adults [3, 4]. The demand of shift work has increased in China as a consequence of rapid industrialization and urbanization. It has been estimated that at least 30% of employees in Asia are involved in performing shift work [5, 6]. Previous epidemiological studies have provided evidence that shift work may be a contributing factor in overweight or obesity, but a majority of these studies were conducted in Western populations [7–9], and few studies have examined these associations in Chinese populations.

Circadian misalignment has previously been implicated as a mechanism underlying the association between shift work and obesity [10–12]. Among the major types of shift work, irregular shifts are thought to be the most detrimental to the alignment of circadian rhythms since an irregular working schedule can induce acute sleep disruption, thereby leading to chronic circadian misalignment [13]. Although several epidemiological studies from different parts of the world have reported a positive association between shift work and the risk of overweight or obesity (classified by body mass index, BMI), the evidence tended to be limited when specific types of shift work were further considered, especially for the association with irregular night shift work [14]. Abdominal obesity, which is an important risk factor of cardiovascular diseases in Western and Asian populations, has been reported to be associated with night shift work [15–17]. However, these findings have been controversial [18, 19]. Therefore, we conducted this study to evaluate the associations between specific types of shift work and different indices of obesity according to the baseline information from a prospective cohort study in China.

## Methods

### Recruitment of participants and data collection

This was a cross-sectional study of Chinese workers who were prospectively recruited in 2013. The subjects were from five companies located in South China, including a nuclear power plant, a steel structure production plant, a semiconductor production company, a printing house, and a beer factory, with a prevalence of night shift workers of 36%, 73%, 84%, 90% and 89%, respectively. These companies were chosen because they had low attrition rates, thereby reducing the likelihood of loss of follow-up. A total of 4,031 employees were invited to participate in the baseline survey. Of these employees, 3,950 consented to participate and completed the baseline questionnaires. The response rate was 98%. In addition, 79 participants who had diagnoses with depression or severe chronic diseases (including renal failure, active hepatitis, cirrhosis, myocardial infraction, chronic obstructive pulmonary disease and cancer) were excluded from this study because these conditions are known to influence body weight. Therefore, a total of 3,871 participants were included in the data analysis.

A self-administered questionnaire was used to collect information on lifetime occupational history, socio-demographics, habits of smoking and alcohol drinking, dietary habits, leisure-time physical activity, sleep patterns and mental stress. The questionnaire was designed for a participant who has a fifth-grade reading level. Trained interviewers with a medical background were available in the interview room to assist the participants in completing the questionnaires if necessary. An occupational health examination was conducted to collect each participant’s anthropometric data and other clinical information. Anthropometric data included the body height (m), body weight (kg) and waist and hip circumference (cm) for each participant and were measured twice by trained health professionals while the participants were wearing light clothes. Waist circumference was measured around the body at the top of the hipbone of each participant immediately after expiration. The anatomical landmark used for this measurement was situated 2.5 cm above the umbilicus.

### Exposure assessment of shift work

Each participant was asked to report a lifetime working history based on a self-administered questionnaire, including information on the company’s name, job title, time schedule of the work, number of years of shift work, and daily working hours for each job. Night shift workers referred to those employees whose work schedules involved a work period between midnight and 6 am at least once per month for more than one year; otherwise, the employees were classified as daytime workers. Night shift workers who had stopped working a shift work schedule for more than one year at the time of the interview were defined as ‘previous night shift workers’, while those who had stopped working a shift work schedule within one year were still regarded as ‘current night shift workers’. Among the current night shift workers, their shift work characteristics were described as ‘permanent night shift work’, ‘rotating shift work’ or ‘irregular shift work’. Permanent night shift workers were defined as those who worked only during the nighttime. Rotating shift workers were those who worked a fixed pattern of daytime and nighttime shifts (e.g., a morning shift, followed by an evening shift, and then a night shift, which was then repeated). Irregular shift workers were those who performed night shift work that was not on a fixed schedule. This type of work mostly occurred for employees who handled emergencies or who had on-call duties. We categorized the number of years of night shift work into 3 subgroups in equal intervals (i.e., ‘<5 years’, ‘5–10 years’, and ‘≥10 years’).

### Assessment of overweight, obesity, and abdominal obesity

Body mass index (BMI) was calculated by body weight (kg) / [body height (m)]^2^. According to the WHO’s definition and for comparability with other Asian studies, overweight and obesity were defined as BMI≥25 kg/m^2^ and BMI≥30 kg/m^2^, respectively, while non-obese participants were referred to those who had a BMI of less than 25 kg/m^2^ [20]. Overweight and obesity were also defined according to the special cut-offs of BMI≥24 kg/m^2^ and BMI≥28 kg/m^2^, respectively, for the Chinese population [21]. Abdominal obesity was defined as a waist circumference >85 or >80 centimeters (cm) for men and women, respectively, according to the recommended criteria for Chinese populations [21].

### Socio-demographic and behavioral characteristics and other factors

Socio-demographic information (i.e., age at interview, sex, education level and marital status) was collected by the self-administered questionnaire. Behavioral factors, including smoking habits, alcohol drinking habits and leisure-time physical activity of each participant, were also assessed based on standardized questions. Smoking status was classified as ‘non-smoker’ ‘current smoker’ or ‘former smoker’. A current smoker was defined as a participant who had smoked at least one cigarette per day for more than six months by the time of the interview. A former smoker was defined as a participant who had quit cigarette smoking for at least 6 months at the time of interview; otherwise, he or she was considered to be a current smoker. Alcohol drinking habits were classified as ‘non-drinker’, ‘current drinker’ or ‘former drinker’. A current drinker was defined as a participant who had consumed alcohol at least once per week for more than six months at the time of interview, and a former drinker was defined as a participant who had stopped consuming alcohol for more than 6 months at the time of interview. Modified Block Food Frequency Questionnaire was used to collect diet information. The consumption of fruits and vegetables was assessed by intake frequency. The items of consumption frequency were grouped as ‘frequent (more than 1 times per day)’ and ‘infrequent (less than 1 times per day)’. Information on leisure-time physical activity was collected by asking the following question: ‘Do you engage in regular physical exercise (≥1 time/week) during leisure time for greater than 20 minutes each time?’ The difference between the time that participants went to bed and the time that they woke up was collected to calculate their daily sleep hours, which were further divided into two groups (‘≥8 hours per day’ and ‘<8 hours per day’). Sleep quality was assessed according to participants’ self-ratings on if their sleep quality at night was ‘good’, ‘fair’, ‘bad’, or ‘extremely bad, requiring sleep pills’. Mental stress was assessed by asking the participants a total of 7 questions that assessed whether they had symptoms of mental disorders, and mental stress levels were further categorized as ‘low level (score<2)’, ‘median level (score 2–5)’ or ‘high level (score≥5)’. Daily working hours were reported by each participant and were further classified into two subgroups (‘≤40 hours/week’ and ‘> 40 hours/week’).

### Statistical analysis

The chi-square test and Student’s *t-*test were used to analyze categorical and continues variables, respectively. The Kolmogorov–Smirnov test was used to examine the normality of age at the time of interview, and the distribution tended to be skewed. The rank-sum test was then employed to test the age difference between the daytime workers and the night shift workers. An unconditional multiple logistic regression model was used to calculate the odds ratio (OR) and the 95% confidence interval (95% CI) of overweight, obesity and abdominal obesity associated with the different types of shift work by adjusting for potential confounding factors (age at interview, sex, marital status, education level, smoking status, alcohol drinking habits, fruit and vegetable consumption, leisure-time physical activity, sleep duration, long working hours, sleep quality and mental stress). These potential confounders were included in the multiple logistic regression model. Furthermore, the association of obesity outcomes with the number of years of night shift work and the number of night shifts per week were examined. The exposure-response relationship of years of night shift work (0, <5, 5–10, ≥10 years) was examined by tests for trends [22]. *p* < 0.05 (two-tailed test) was considered statistically significant.

Sensitivity analysis was performed to examine the robustness of the results among male workers with the Chinese-specific BMI cut-offs of 24 kg/m^2^ and 28 kg/m^2^ for overweight and obesity, respectively [21]. We hypothesized that those performing night shift work, in particular, the irregular type, would be more likely to develop abdominal obesity with a higher body mass index. Therefore, we further defined an additional category of **‘**BMI≥25 kg/m^2^ and waist circumference >85 cm or 80 cm for men or women’ to examine whether workers who engaged in irregular shift work were more prone to this special type of obesity status than the daytime workers. To estimate the effect scale of leisure-time physical activities in regression model, we examined the odds ratios in the regression model that removing the variable of leisure-time physical activities. All analyses were performed using SPSS software (version 22.0, SPSS Inc., Chicago, IL, USA).

### Ethics

This study was approved by the Joint Chinese University of Hong Kong—New Territories East Cluster Clinical Research Ethics Committee (CRE-2013.107). All participants provided written informed consent.

## Results

### Characteristics of participants

Of the 3,871 participants (aged from 18 to 61 years) with complete data regarding night shift work exposure and anthropometric measurements, a total of 1,039 (26.8%) workers were overweight, and 83 (2.1%) workers were obese, while 1,673 (43.2%) workers had abdominal obesity. For the daytime workers, 496 (29.6%) workers were overweight, 39 (2.3%) were obesity and 823 (49.1%) were abdominal obesity. Among the current night shift workers, the corresponding prevalence was 22.0% for overweight, 1.8% for obesity and 36.9% for abdominal obesity. The distribution of night shift workers and overweight/obesity among working site was shown in S1 Table. Compared with night shift workers (Table 1), daytime workers were relatively older (median age: 30 vs. 28 years old) and more likely to smoke, drink alcohol and be obese, but they were also more likely to spend time doing physical activity and had shorter working hours.

**Table 1:** Selected baseline characteristics among Chinese workers in a prospective cohort study stratified by night shift work schedules.

Variables	Daytime workers	Previous night shift workers	Current night shift workers
	N (%)	N (%)	N (%)
No. of participants	1,677 (43.4)	437 (11.3)	1,757 (45.4)
Age at interview, years (median/ range)	30/18-61	33/18-59	28/18-59*
Sex			
Male	1,605 (95.7)	431 (98.6)	1,395 (79.5)*
Female	72 (4.3)	5 (1.4)	359 (20.5)
Education			
Primary or below	3 (0.2)	1 (0.2)*	5 (0.3)*
Junior high school	34 (2.0)	46 (10.6)	367 (21.1)
Senior high school	221 (13.2)	313 (72.1)	482 (27.7)
College or above	1,410 (84.5)	74 (17.1)	885 (50.9)
Marital status			
Single	527 (31.5)	113 (26.0)	806 (46.1)*
Married	1,124 (67.3)	317 (72.9)	930 (53.2)
Divorced	20 (1.2)	5 (1.1)	12 (0.7)
Smoking status			
Non-smoker	1,182 (71.0)	218 (50.6)	1,340 (77.2)*
Current smoker	390 (23.4)	184 (42.7)	362 (20.9)
Former smoker	93 (5.6)	29 (6.7)	34 (2.0)
Alcohol drinking			
Non-drinker	1,244 (74.7)	230 (52.8)	1,381 (79.8)*
Current drinker	405 (24.3)	200 (45.9)	340 (19.6)
Former drinker	16 (1.0)	6 (1.4)	10 (0.6)
Leisure-time physical activity			
≤1 time/week	559 (33.3)	181 (41.4)	754 (42.9)*
>1 time/week	1,118 (66.7)	256 (58.6)	1,003 (57.1)
Food consumption (fruits)			
Infrequent	403 (24.0)	63 (14.4)	380 (21.6) *
Frequent	1274 (76.0)	374 (85.6)	1377 (78.4)
Food consumption (vegetables)			
Infrequent	1129 (67.3)	275 (62.9)	934 (53.2) *
Frequent	548 (32.7)	162 (37.1)	823 (46.8)
Sleep duration			
6–8 hours per day	1,074 (72.6)	321 (77.9)	868 (58.0)*
≥8 hours per day	43 (2.9)	17 (4.1)	34 (2.3)
<6 hours per day	363 (24.5)	74 (18.0)	595 (39.7)
Sleep quality			
Good	730 (44.1)	122 (28.4)	572 (33.7)*
Fair	836 (50.5)	246 (57.2)	991 (58.4)
Bad	84 (5.1)	56 (13.0)	127 (7.5)
Extremely bad	6 (0.4)	6 (1.4)	7 (0.4)
Working hours			
≤40 hours/week	1,191 (71.0)	418 (95.7)	768 (43.7)*
>40 hours/week	486 (29.0)	19 (4.3)	989 (56.3)
Mental stress			
Low level	1,325 (79.0)	304 (69.6)	1394 (79.3)
Middle level	323 (19.3)	116 (26.5)	326 (18.6)
High level	29 (1.7)	17 (3.9)	37 (2.1)
Obese status ^a^			
Non-obesity	1,142 (68.1)	267 (61.1)	1,340 (76.3)*
Overweight	496 (29.6)	157 (35.9)	386 (22.0)
Obesity	39 (2.3)	13 (3.0)	31 (1.8)
Abdominal obesity	823 (49.1)	202 (46.2)	648 (36.9)*

^a^ Non-obesity: body mass index (BMI) <25 kg/m^2^; overweight, BMI 25–30 kg/m^2^; obesity: BMI≥30 kg/m^2^; abdominal obesity: waist circumference >85 cm for men or >80 cm for women

* Comparing to daytime workers, *p* <0.05.

### Shift work patterns, overweight and obesity

A total of 1,757 employees (45.4% of all participants) were night shift workers with an average of 4.76 years of exposure to night shift work. Although the proportions of overweight and obesity in night shift workers were lower than those in the daytime workers, a positive association between night shift work and obesity was observed but did not reach statistical significance (OR = 1.17, 95% CI, 0.97–1.41) after adjusting for potential confounding factors (Table 2). Further subgroup analysis by the type of night shift work showed that permanent night shift work had the strongest association with overweight status (OR = 3.94, 95% CI, 1.40–11.03), followed by irregular night shift work (OR = 1.56, 95% CI, 1.13–2.14). Moreover, no significant association between rotating night shift work and overweight or obesity was noted. An exposure-response relationship with obesity outcomes revealed a significantly positive gradient between overweight and the number of years of night shift work (test for trend, *p* = 0.014) and the number of night shifts (OR = 1.19, 95% CI, 1.04–1.37, for every increment of night shifts worked per week).

**Table 2:** Odds ratios (ORs) and 95% confidence intervals (95% CIs) for the association between the different types of night shift work and obese outcomes obtained from the baseline survey of 3,871 Chinese workers.

Characteristics				BMI<25 kg/m^2^	BMI≥25 kg/m^2^		BMI≥30 kg/m^2^	
				N (%)	N (%)	Adjusted OR* (95% CI)	N (%)	Adjusted OR* (95% CI)
No. of participants				2749 (100.0)	1039 (100.0)	—	83 (100.0)	—
Types of shift work ^a^								
	Daytime work			1,142 (41.5)	496 (47.7)	1.00	39 (47.0)	1.00
	Night shift work			1607 (58.5)	543 (52.3)	1.17 (0.97–1.41)	44 (53.0)	1.27 (0.74–2.18)
		Previous night shift work		267 (9.7)	157 (15.1)	1.33 (0.99–1.79)	13 (15.7)	1.17 (0.51–2.69)
		Current night shift work		1,340 (48.7)	386 (37.2)	1.12 (0.92–1.37)	31 (37.3)	1.30 (0.72–2.38)
			Permanent night shift	14 (0.5)	11 (1.1)	3.94 (1.40–11.03)	0 (0.0)	NA ^e^
			Rotating night shift	1,127 (41.0)	278 (26.8)	0.95 (0.76–1.19)	22 (26.5)	1.12 (0.56–2.24)
			Irregular night shift	199 (7.2)	97 (9.3)	1.56 (1.13–2.14)	9 (10.8)	2.41 (0.99–5.84)
Years of night shift work ^b^ ^c^								
			Daytime work	1,142 (41.5)	496 (47.7)	1.00	39 (47.0)	1.00
			<5 years	1,095 (39.8)	253 (24.4)	0.85 (0.60–1.22)	26 (31.3)	1.77 (0.71–4.40)
			5–10 years	286 (10.4)	139 (13.4)	1.11 (0.75–1.63)	8 (9.6)	0.37 (0.08–1.74)
			≥10 years	226 (8.2)	151 (14.5)	1.25 (0.82–1.92)	10 (12.0)	1.21 (0.36–4.04)
			*p value (test for trend)*			*0*.*014*		*0*.*732*
				Mean±SD	Mean±SD	Adjusted OR* (95% CI)	Mean±SD	Adjusted OR* (95% CI)
Years engaged in night shift work ^c^				4.15±5.19	6.60±6.05	1.02 (0.99–1.37)	7.77±7.87	1.02 (0.97–1.08)
Nights of shifts per week ^d^				1.24±0.81	1.46±1.21	1.19 (1.04–1.37)	1.28±0.38	1.35 (0.92–1.99)

* Model 1: In addition to the type of shift work, the variables included in Model 1 are age at interview, sex, marital status, education level, smoking status, drinking habits, consumption of fruit and vegetables, leisure-time physical activity, sleep duration, sleep quality, working hours and mental stress

^a^ Using daytime work as a reference group

^b^ Using shift work year = 0 as a reference group

^c^ The variable ‘night shifts per week’ is also included in Model 1

^d^ The variable “years engaged in night shift work” is also included in Model 1

^e^ NA: not applicable, as the calculation was not possible due to no cases in this group.

### Shift work patterns and abdominal obesity

Night shift work had a significant impact on the prevalence of abdominal obesity (OR = 1.20, 95% CI, 1.01–1.43), with a significant association with permanent night shift work (OR = 3.34, 95% CI, 1.19–9.37) (Table 3).

**Table 3:** Odds ratios (ORs) and 95% confidence intervals (95% CIs) for the association between the different types of night shift work and abdominal obesity in 3,871 Chinese workers.

Characteristics			Non-abdominal obesity	Abdominal obesity	
			N (%)	N (%)	Adjusted OR* (95% CI)
**No. of Participants**			2,198 (100.0)	1673 (100.0)	—
**Types of shift work ^a^**					
	Daytime work		854 (38.9)	823 (49.2)	1.00
	Night shift work		1344 (61.1)	850 (50.8)	1.20 (1.01–1.43)
		Previous night shift work	235 (10.7)	202 (12.1)	1.09 (0.82–1.45)
		Current night shift work	1,109 (50.5)	648 (38.7)	1.23 (1.03–1.46)
		Permanent night shift	11 (0.5)	14 (0.8)	3.34 (1.19–9.37)
		Rotating night shift	933 (42.4)	494 (29.5)	1.17 (0.96–1.44)
		Irregular night shift	165 (7.5)	140 (8.4)	1.26 (0.94–1.69)
**Years of night shift work ^b^^c^**					
		Daytime work	854 (38.9)	823 (49.2)	1.00
		<5 years	964 (43.9)	410 (24.5)	0.95 (0.69–1.30)
		5–10 years	224 (10.2)	209 (12.5)	0.88 (0.60–1.28)
		≥10 years	156 (7.1)	231 (13.8)	1.15 (0.73–1.80)
		*p value (test for trend)*			*0*.*064*
			Mean±SD	Mean±SD	Adjusted OR* (95% CI)
**Years engaged in night shift work ^c^**			3.57±4.72	6.79±6.23	1.04 (1.01–1.06)
**Nights of shifts per week ^d^**			1.25±0.80	1.38±1.09	1.09 (0.95–1.24)

Non-abdominal obesity, waist circumference ≤85 cm for men or ≤80 cm for women; Abdominal obesity, waist circumference >85 cm for men or >80 cm for women

* Model 1: In addition to the type of shift work, the variables included in Model 1 were age at interview, sex, marital status, education level, smoking status, drinking habits, consumption of fruit and vegetables, leisure-time physical activity, sleep duration, sleep quality, working hours and mental stress

^a^ Using daytime work as a reference group

^b^ Using shift work year = 0 as a reference group

^c^ The variable ‘night shifts per week’ was also included in Model 1

^d^ The variable “years engaged in night shift work” was also included in Model 1.

### Sensitivity analysis

Sensitivity analyses (S2 Table) showed that the association between night shift work and the specifically defined obese outcome ‘BMI≥25 kg/m^2^ and abdominal obesity’ were similar to those presented in Table 3. Similar results were obtained when specific BMI cut-offs of 24 kg/m^2^ for overweight and 28 kg/m^2^ for obesity for Chinese populations were adopted, and similar results were observed after the female workers were excluded from the data analyses (S3, S4 and S5 Tables). Sensitivity analysis was conducted by removing the variable of leisure-time physical activities from the model, the odds ratios were almost unchanged (S6 and S7 Tables).

## Discussion

### Summary of the main results of this study

Our study demonstrated that permanent was positively associated with an increased prevalence of overweight or abdominal obesity among Chinese workers. A possible association was observed between irregular night shift work and overweight. A positive exposure-response relationship was observed between the intensity of night shift work exposure (e.g., number of years of night shift work or number of night shifts per week) and the prevalence of overweight or abdominal obesity.

The results from our study of Chinese workers are consistent with the findings from previous epidemiological studies that reported a possible gradient effect of night shift work on overweight or weight gain [23–28]. Indeed, occupational studies in Taiwan and Japan reported that shift workers had an excess risk ranging from 6% to 170% for increases in BMI or being obese compared with daytime workers [16, 29]. Interestingly, Asians tended to have less muscle but more abdominal fat than Caucasians with the same BMI [30]. However, few studies have examined the relationship between shift work and abdominal obesity. In 2010, Chen et al. reported a positive association between night shift work and abdominal obesity, with an OR of 2.9 (95% CI, 1.7–5.1) in Taiwan [16], which is consistent with our findings.

### Comparisons with previous studies

Rotating shifts are the major type of shift work in many industrial sectors. Although there are several studies that have indicated that rotating shift work plays a hazardous role in the etiology of obesity [28, 31, 32], De Bacquer et al. and Karlsson et al. reported a weak association between rotating night shift work and obesity that was not statistically significant [18, 19]. Other epidemiological studies failed to find a relationship between rotating shift work and obesity [29, 33, 34]. Evidence regarding whether permanent or irregular night shift workers experience a higher risk of obesity than other workers is lacking. Until now, only a small study of 57 Brazilian male truck drivers (including 31 irregular shift workers) reported that the BMIs among irregular shift workers were higher than those of daytime workers [14], a finding that was replicated in our study. Compared with rotating night shift workers, irregular night shift workers need to work harder to adapt to their working schedules, which causes a higher intensity of circadian misalignment [13, 35]. In our study, permanent night shift workers showed the highest odds ratio for the prevalence of overweight or abdominal obesity. However, given that the number of permanent night shift workers was small, this finding should be interpreted with caution. Another epidemiological study involving over 20,000 employees published in 2010 also reported that permanent night shift workers had a higher risk of obesity than rotating night shift workers [34]. The mechanisms for the development of obesity among shift workers may vary. For example, permanent night shift workers have strict nocturnal working hours that may lead them to have less motivation [36] and more barriers to maintain adequate physical exercise during their leisure time [37]. These behavioral factors may play greater roles than circadian misalignment in the development of obesity among permanent night shift workers.

### Interpretations of the potential pathogenic mechanisms

The association between night shift work and the development of obesity can be explained by different mechanisms [38]. Shift work schedules can lead to desynchronization between the workers’ sleep-wake cycle and the natural light-dark cycle [39], which may consequently result in misalignment between sleeping and waking or feeding and fasting patterns [10]. Shift workers are exposed to artificial light during their night working period, which may suppress the secretion of melatonin [40]. Melatonin is an important hormone that sustains the circadian oscillation of many peripheral metabolic-related hormones, including insulin, cortisol and leptin [41]. If the normal diurnal variation of one or more of these hormones is lost, this may disrupt the balance of energy metabolism and lead to weight gain [10, 42–45]. Moreover, shift work may lead to lifestyle changes, including sleep deficiency, and decreased physical activity during leisure time, all of which have been linked to weight gain and obesity [46]. In our study, we found a positive effect of night shift work with obesity after adjusting sleep information, food consumption and leisure-time physical activities in regression model. And the positive association between night shift work and obesity remain almost unchanged after removing the variable of leisure-time physical activities. Those results reveal that the BMI/ body shape difference between daytime and night shift workers not only due to the different patterns of eating habits and physical activities, circadian disruption could affect obesity development prominently.

### Strengths and limitations

Our study has several strengths. First, this study included a large number of Chinese shift workers in the sample. Second, detailed night shift work schedules were assessed. Third, important lifestyle factors were determined. Fourth, the effects of specific types of night shift work on different obesity outcomes were compared in the same study for the first time in a Chinese population.

However, several limitations should be mentioned. First, the cross-sectional study design might raise concern for examining the risk factors; therefore, the results from the baseline survey need to be confirmed in future prospective cohort studies. Second, a ‘healthy night worker effect’ might be another concern, as susceptible or unhealthy night shift workers might have quit or changed their jobs, leading to an underestimation of the effects of interest; however, the participants in this study were relatively young. Therefore, it was less likely that they left their positions due to illnesses that occur with aging. The sensitivity analysis showed that the results remained unchanged after excluding the 11.3% of workers with previous night shift work experience. Third, the shift work schedules may be different among the companies, which may have led to variation within the same types of night shift work, particularly for the rotating shift and irregular shift subgroups. However, this potential misclassification is likely to be non-differential and may have caused a dilution of the association with rotating or irregular night shift work. Fourth, information on the workers’ night shift work history was collected by a self-administered questionnaire, so recall bias may be a concern. We compared the shift work schedules reported by the workers with those kept in the company records and the agreement was high, which indicates that recall bias, if it is present, should be low. Fifth, we did not include other occupational hazards in the regression model in this study; however, the occupational hygiene inspection data showed that the studied companies had good compliance with the occupational exposure limits of China. Sixth, the distribution of some important covariates and confounding factors such as age, gender, marital status and education level were uneven because of the inherent limitation of cluster sampling. We did multivariate and sensitivity analysis to minimize their potential effect and robust our conclusion. However, the consistent effect estimates on overweight/ obesity of permanent night shift were based on limited sample size, that also considered as a limiting aspect. Finally, the occupational physical activities of employees were not collected, although the working hours of each participant were adjusted as a surrogate of workload. Also, we did not collect the amount of food consumption as our pilot test showed that the recall of food amount was not reliable. We are aware that not including food amount in the model may introduce residual confounding to obesity outcome, and we therefore addressed this as one of the limitation of this study. The results from this baseline survey need to be replicated in our ongoing prospective cohort study by recruiting more participants with longer follow-up durations.

In conclusion, this study demonstrates that night shift work, particularly permanent night shift work associated with increased risk of overweight among the Chinese population. Permanent and irregular night shift work associate with the increased risk of abdominal obesity but need to be further verified. Results from this study suggest that implementation of an optimized shift work schedule via effective administration control is warranted.

## Supporting information

S1 Table Distribution of night shift workers and overweight/obesity among 5 study sites. (DOCX)

S2 Table Odds ratios (ORs) and 95% confidence intervals (95% CIs) for the associations between different types of night shift work and an additional obese outcome ‘BMI≥25 kg/m^2^ and abdominal obesity’. (DOCX)

S3 Table Odds ratios (ORs) and 95% confidence intervals (95% CIs) for the associations between different types of night shift work and obese outcomes obtained from the baseline survey using the specific BMI cut-offs of 24 kg/m^2^ for overweight and 28 kg/m^2^ for obesity for Chinese populations. (DOCX)

S4 Table Odds ratios (ORs) and 95% confidence intervals (95% CIs) for the associations between different types of night shift work and obese outcomes obtained from the baseline survey among male workers. (DOCX)

S5 Table Odds ratios (ORs) and 95% confidence intervals (95% CI) for the associations between different types of night shift work and abdominal obesity among Chinese male workers. (DOCX)

S6 Table Odds ratios (ORs) (removed leisure-time physical activities) and 95% confidence intervals (95% CIs) for the association between the different types of night shift work and obese outcomes obtained from the baseline survey of 3,871 Chinese workers. (DOCX)

S7 Table Odds ratios (ORs) (removed leisure-time physical activities) and 95% confidence intervals (95% CIs) for the association between the different types of night shift work and abdominal obesity in 3,871 Chinese workers. (DOCX)

## Abbreviations

OR: odds ratio
95% CI: 95% confidence interval
BMI: body mass index
