# Supplementary material for: Night shift work exposure profile and obesity: Baseline results from a Chinese night shift worker cohort
Source: PLoS One. 2018 May 15;13(5):e0196989. doi: 10.1371/journal.pone.0196989 (PMC5953447; doi:10.1371/journal.pone.0196989)
Supplement: S1 Table — (DOCX) [file pone.0196989.s001.docx]

S1 Table. Distribution of night shift workers and overweight/obesity among 5 study sites

| Study site | A | B | C | D | E |
| --- | --- | --- | --- | --- | --- |
| Number of workers | 506 | 2214 | 248 | 344 | 559 |
| N% of night shift workers in the site | 73% | 36% | 84% | 89% | 90% |
| Distribution of night shift workers | 17.1% | 36.2% | 9.5% | 14.0% | 23.2% |
| Prevalence of overweight (BMI 25-29.9) | 35.8% | 29.7% | 8.9% | 22.1% | 18.4% |
| Prevalence of obesity (BMI≥30) | 3.8% | 2.3% | 1.2% | 1.5% | 0.9% |
