# Supplementary material for: Night shift work exposure profile and obesity: Baseline results from a Chinese night shift worker cohort
Source: PLoS One. 2018 May 15;13(5):e0196989. doi: 10.1371/journal.pone.0196989 (PMC5953447; doi:10.1371/journal.pone.0196989)
Supplement: S2 Table — (DOCX) [file pone.0196989.s002.docx]

S2 Table. Odds ratios (ORs) and 95% confidence intervals (95% CIs) for the associations between different types of night shift work and the additional obese outcome “BMI≥25 kg/m^2^ and abdominal obesity”

| Characteristics | | | | BMI<25 kg/m^2^ |  | Abdominal obesity and BMI≥25 kg/m^2^ | |
| --- | --- | --- | --- | --- | --- | --- | --- |
|  |  |  |  | N (%) |  | N (%) | Adjusted OR^*^ (95% CI) |
| No. of participants | | | | 2749 (100.0) |  | 999 (100.0) | -- |
| Types of shift work ^a^ | | | |  |  |  |  |
|  | Daytime work | | | 1142 (41.5) |  | 483 (48.3) | 1.00 |
|  | Night shift work | | | 1607 (58.5) |  | 516 (51.7) | 1.20 (0.99-1.44) |
|  | | Previous night shift work | | 267 (9.7) |  | 150 (15.0) | 1.31 (0.98-1.77) |
|  | | Current night shift work | | 1340 (48.7) |  | 366 (36.6) | 1.16 (0.96-1.42) |
|  | | | Permanent night shift | 11 (0.4) |  | 9 (0.9) | 3.68 (1.25-10.86) |
|  | | | Rotating night shift | 1127 (41.0) |  | 262 (26.2) | 1.03 (0.82-1.29) |
|  | | | Irregular night shift | 199 (7.2) |  | 95 (9.5) | 1.58 (1.15-2.17) |
| Years of night shift work ^b c^ | | | |  |  |  |  |
|  | | Daytime work | | 1142 (41.5) |  | 483 (48.3) | 1.00 |
|  | | <5 years | | 1095 (39.8) |  | 238 (23.8) | 0.97 (0.68-1.38) |
|  | | 5-10 years | | 286 (10.4) |  | 129 (12.9) | 1.06 (0.71-1.57) |
|  | | ≥10 years | | 226 (8.2) |  | 149 (14.9) | 1.29 (0.90-1.59) |
|  | | *p value (test for trend)* | |  |  |  | 0.011 |
|  | | | | Mean±SD |  | Mean±SD | Adjusted OR^*^ (95% CI) |
| Years engaged in night shift work ^c^ | | | | 4.15±5.19 |  | 7.02±6.29 | 1.02 (1.00-1.05) |
| Nights of shifts per week ^d^ | | | | 1.24±0.81 |  | 1.46±1.18 | 1.20 (1.04-1.38) |

* Model 1: In addition to the types of night shift work, the variables included in Model 1 were age at interview, gender, marital status, education level, smoking status, drinking habits, consumption of fruit and vegetables, leisure-time physical activity, sleep duration, sleep quality, working hours and mental stress; ^a^ Using daytime work as a reference group; ^b^ Using shift work year=0 as a reference group; ^c^ The variable “night shifts per week” was also included in Model 1; ^d^ The variable “ years engaged in night shift work” was also included in Model 1.
