# Supplementary material for: Night shift work exposure profile and obesity: Baseline results from a Chinese night shift worker cohort
Source: PLoS One. 2018 May 15;13(5):e0196989. doi: 10.1371/journal.pone.0196989 (PMC5953447; doi:10.1371/journal.pone.0196989)
Supplement: S3 Table — (DOCX) [file pone.0196989.s003.docx]

S3 Table. Odds ratios (ORs) and 95% confidence intervals (95% CIs) for the associations between different types of night shift work and obese outcomes obtained from the baseline survey using the specific BMI cut-offs of 24 kg/m^2^ for overweight and 28 kg/m^2^ for obesity for Chinese populations

| Characteristics | | | | BMI<24 kg/m^2^ |  | BMI≥24 kg/m^2^ | |  | BMI≥28 kg/m^2^ | |
| --- | --- | --- | --- | --- | --- | --- | --- | --- | --- | --- |
|  |  |  |  | N (%) |  | N (%) | Adjusted OR^*^ (95% CI) |  | N (%) | Adjusted OR^*^ (95% CI) |
| No. of participants | | | | 2293 (100.0) |  | 1317 (100.0) | -- |  | 261 (100.0) | -- |
| Types of shift work ^a^ | | | |  |  |  |  |  |  |  |
|  | Daytime work | | | 932 (40.6) |  | 624 (47.4) | 1.00 |  | 121 (46.4) | 1.00 |
|  | Night shift work | | | 1361 (59.4) |  | 693 (52.6) | 1.23 (1.03-1.47) |  | 140 (53.6) | 1.36 (0.98-1.89) |
|  | | Previous night shift work | | 220 (9.6) |  | 171 (13.0) | 1.29 (0.96-1.74) |  | 46 (17.6) | 1.56 (0.95-2.58) |
|  | | Current night shift work | | 1141 (49.8) |  | 522 (39.6) | 1.21 (1.00-1.46) |  | 94 (36.0) | 1.29 (0.90-1.85) |
|  | | | Permanent night shift | 12 (0.5) |  | 12 (0.9) | 3.87 (1.34-11.18) |  | 1 (0.4) | 1.21 (0.08-17.59) |
|  | | | Rotating night shift | 964 (42.0) |  | 397 (30.1) | 1.06 (0.86-1.31) |  | 66 (25.3) | 1.01 (0.67-1.54) |
|  | | | Irregular night shift | 165 (7.2) |  | 113 (8.6) | 1.43 (1.05-1.95) |  | 27 (10.3) | 1.91 (1.10-3.33) |
| Years of night shift work ^b c^ | | | |  |  |  |  |  |  |  |
|  | | | Daytime work | 932 (40.6) |  | 624 (47.4) | 1.00 |  | 121 (46.4) | 1.00 |
|  | | | <5 years | 968 (42.2) |  | 337 (25.6) | 0.93 (0.66-1.31) |  | 69 (26.4) | 1.16 (0.64-2.09) |
|  | | | 5-10 years | 235 (10.2) |  | 167 (12.7) | 0.96 (0.65-1.43) |  | 31 (11.9) | 0.76 (0.37-1.57) |
|  | | | ≥10 years | 158 (6.9) |  | 189 (14.4) | 1.07 (0.67-1.71) |  | 40 (15.3) | 1.31 (0.60-2.84) |
|  | | | *p value (test for trend)* |  |  |  | 0.002 |  |  | 0.041 |
|  | | | | Mean±SD |  | Mean±SD | Adjusted OR^*^ (95% CI) |  | Mean±SD | Adjusted OR^*^ (95% CI) |
| Years engaged in night shift work ^c^ | | | | 3.67±4.85 |  | 6.70±6.08 | 1.02 (1.00-1.05) |  | 7.15±6.64 | 1.04 (1.00-1.07) |
| Nights of shifts per week ^d^ | | | | 1.23±0.76 |  | 1.43±1.19 | 1.18 (1.03-1.36) |  | 1.36±0.94 | 1.17 (0.91–1.51) |

^*^ Model 1: In addition to the types of night shift work, the variables included in Model 1 were age at interview, gender, marital status, education level, smoking status, drinking habits, consumption of fruit and vegetables, physical activity, sleep duration, sleep quality, working hours and mental stress; ^a^ Using daytime work as a reference group; ^b^ Using shift work year=0 as a reference group; ^c^ The variable “night shifts per week” was also included in Model 1; ^d^ The variable “years engaged in night shift work” was also included in Model 1.
