# Supplementary material for: Night shift work exposure profile and obesity: Baseline results from a Chinese night shift worker cohort
Source: PLoS One. 2018 May 15;13(5):e0196989. doi: 10.1371/journal.pone.0196989 (PMC5953447; doi:10.1371/journal.pone.0196989)
Supplement: S4 Table — (DOCX) [file pone.0196989.s004.docx]

S4 Table. Associations between the different types of night shift work and baseline bodyweight among male workers

| Characteristics | | | | BMI<24 kg/m^2^ |  | BMI≥25 kg/m^2^ | |  | BMI≥30 kg/m^2^ | |
| --- | --- | --- | --- | --- | --- | --- | --- | --- | --- | --- |
|  |  |  |  | N (%) |  | N (%) | Adjusted OR^*^ (95% CI) |  | N (%) | Adjusted OR^*^ (95% CI) |
| No. of participants | | | | 2387 (100.0) |  | 967 (100.0) | -- |  | 77 (100.0) | -- |
| Types of shift work ^a^ | | | |  |  |  |  |  |  |  |
|  | Daytime work | | | 1087 (45.5) |  | 482 (49.8) | 1.00 |  | 36 (46.8) | 1.00 |
|  | Night shift work | | | 1300 (50.2) |  | 485 (50.2) | 1.18 (0.98-1.42) |  | 41 (53.2) | 1.14 (0.93-1.39) |
|  | | Previous night shift work | | 264 (11.1) |  | 154 (15.9) | 1.30 (0.96-1.39) |  | 13 (16.9) | 2.89 (0.62-13.42) |
|  | | Current night shift work | | 1036 (43.4) |  | 331 (34.2) | 1.14 (0.93-1.39) |  | 28 (36.4) | 1.46 (0.84-2.55) |
|  | | | Permanent night shift | 12 (0.5) |  | 10 (1.0) | 4.62 (1.57-13.60) |  | 0 (0.0) | NA |
|  | | | Rotating night shift | 838 (35.1) |  | 230 (23.8) | 0.97 (0.77-1.21) |  | 19(24.6) | 1.42 (0.70-2.86) |
|  | | | Irregular night shift | 186 (7.8) |  | 91 (9.4) | 1.59 (1.15-2.20) |  | 9 (15.5) | 2.95 (1.21-7.21) |
| Years of night shift work ^b c^ | | | |  |  |  |  |  |  |  |
|  | | | Daytime work | 1087 (45.5) |  | 482 (49.8) | 1.00 |  | 36 (46.8) | 1.00 |
|  | | | <5 years | 865 (36.2) |  | 215 (22.2) | 0.86 (0.60-1.22) |  | 24 (31.2) | 1.92 (0.77-4.77) |
|  | | | 5-10 years | 254 (10.6) |  | 131 (13.5) | 1.10 (0.74-1.64) |  | 7 (9.1) | 0.30 (0.60-1.52) |
|  | | | ≥10 years | 181 (7.6) |  | 139 (14.4) | 1.23 (0.78-1.94) |  | 10 (13.0) | 1.33 (0.36-4.94) |
|  | | | *p value (test for trend* |  |  |  | 0.008 |  |  | 0.615 |
|  | | | | Mean±SD |  | Mean±SD | Adjusted OR^*^ (95% CI) |  | Mean±SD | Adjusted OR^*^ (95% CI) |
| Years engaged in night shift work ^c^ | | | | 4.52±5.00 |  | 7.10±5.66 | 1.03 (1.00-1.05) |  | 8.00±7.34 | 1.03 (0.98-1.09) |
| Nights of shifts per week ^d^ | | | | 1.27±0.82 |  | 1.48±1.23 | 1.21 (1.04-1.40) |  | 1.30±0.38 | 1.59 (1.05-2.40) |

^*^ Model 1: In addition to the types of night shift work, the variables included in Model 1 were age at interview, marital status, education level, smoking status, drinking habits, eating fruit (continuous), eating vegetables (continuous), leisure-time physical activity, sleep duration, sleep quality, working hours, and mental stress; ^a^ Using daytime work as a reference group; ^b^ Using shift work year=0 as a reference group; ^c^ The variable “night shifts per week” was also included in Model 1; ^d^ The variable “years engaged in shift work” was also included in Model 1.
