# Supplementary material for: Night shift work exposure profile and obesity: Baseline results from a Chinese night shift worker cohort
Source: PLoS One. 2018 May 15;13(5):e0196989. doi: 10.1371/journal.pone.0196989 (PMC5953447; doi:10.1371/journal.pone.0196989)
Supplement: S5 Table — (DOCX) [file pone.0196989.s005.docx]

S5 Table. Associations between the different types of night shift work and abdominal obesity among male workers

| Characteristics | | | | Non-abdominal obesity |  | Abdominal obesity | |
| --- | --- | --- | --- | --- | --- | --- | --- |
|  |  |  |  | N (%) |  | N (%) | Adjusted OR^*^ (95% CI) |
| No. of participants | | | | 1881 (100.0) |  | 1550 (100.0) | -- |
| Types of shift work | | | |  |  |  |  |
|  | Daytime work ^a^ | | | 804 (42.7) |  | 801 (51.7) | 1.00 |
|  | Night shift work ^a^ | | | 1077 (57.3) |  | 749 (48.3) | 1.18 (0.99-1.41) |
|  | | Previous night shift work ^a^ | | 231 (12.3) |  | 200 (12.9) | 1.06 (0.79-1.42) |
|  | | Current night shift work ^a^ | | 846 (45.0) |  | 549 (35.4) | 1.22 (1.01-1.46) |
|  | | | Permanent night shift ^a^ | 10 (0.5) |  | 12 (0.8) | 2.91 (1.02-8.34) |
|  | | | Rotating night shift ^a^ | 684 (36.3) |  | 403 (26.0) | 1.14 (0.93-1.40) |
|  | | | Irregular night shift ^a^ | 152 (8.1) |  | 134 (8.6) | 1.25 (0.92-1.68) |
| Years of night shift work ^b c^ | | | |  |  |  |  |
|  | | | Daytime work | 804 (42.7) |  | 801 (51.7) | 1.00 |
|  | | | <5 years | 757 (40.2) |  | 347 (22.4) | 0.92 (0.66-1.27) |
|  | | | 5-10 years | 198 (10.5) |  | 194 (12.5) | 0.86 (0.58-1.27) |
|  | | | ≥10 years | 122 (6.5) |  | 208 (13.4) | 1.25 (0.78-2.02) |
|  | | | *p value (test for trend* |  |  |  | 0.037 |
|  | | | | Mean±SD |  | Mean±SD | Adjusted OR^*^ (95% CI) |
| Years engaged in night shift work ^c^ | | | | 3.75±4.73 |  | 7.01±6.15 | 1.04 (1.01-1.06) |
| Nights of working per week ^d^ | | | | 1.29±0.87 |  | 1.39±1.04 | 1.07 (0.92-1.23) |

Non-abdominal obesity, waist circumference ≤85 cm for men or ≤80 cm for women; Abdominal obesity, waist circumference >85 cm for men or >80 cm for women;

* Model 1: In addition to the types of night shift work, the variables included in Model 1 were age at interview, marital status, education level, smoking status, drinking habits, consumption of fruit and vegetables, leisure-time physical activity, sleep duration, sleep quality, working hours, and mental stress; ^a^ Using daytime work as a reference group; ^b^ Using shift work year=0 as a reference group; ^c^ The variable “night shifts per week” was also included in Model 1; ^d^ The variable “years engaged in night shift work” was also included in Model 1.
