# Supplementary material for: Night shift work exposure profile and obesity: Baseline results from a Chinese night shift worker cohort
Source: PLoS One. 2018 May 15;13(5):e0196989. doi: 10.1371/journal.pone.0196989 (PMC5953447; doi:10.1371/journal.pone.0196989)
Supplement: S6 Table — (DOCX) [file pone.0196989.s006.docx]

S6 Table. Odds ratios (ORs) (removed leisure-time physical activities) and 95% confidence intervals (95% CIs) for the association between the different types of night shift work and obese outcomes obtained from the baseline survey of 3,871 Chinese workers

| Characteristics | | | | BMI<25 kg/m^2^ |  | BMI≥25 kg/m^2^ | |  | BMI≥30 kg/m^2^ | |
| --- | --- | --- | --- | --- | --- | --- | --- | --- | --- | --- |
|  |  |  |  | N (%) |  | N (%) | Adjusted OR^*^ (95% CI) |  | N (%) | Adjusted OR^*^ (95% CI) |
| No. of participants | | | | 2749 (100.0) |  | 1039 (100.0) | -- |  | 83 (100.0) | -- |
| Types of shift work ^a^ | | | |  |  |  |  |  |  |  |
|  | Daytime work | | | 1,142 (41.5) |  | 496 (47.7) | 1.00 |  | 39 (47.0) | 1.00 |
|  | Night shift work | | | 1607 (58.5) |  | 543 (52.3) | 1.17 (0.97-1.40) |  | 44 (53.0) | 1.24 (0.73-2.14) |
|  | | Previous night shift work | | 267 (9.7) |  | 157 (15.1) | 1.33 (0.99-1.79) |  | 13 (15.7) | 1.19 (0.52-2.72) |
|  | | Current night shift work | | 1,340 (48.7) |  | 386 (37.2) | 1.12 (0.92-1.37) |  | 31 (37.3) | 1.27 (0.70-2.29) |
|  | | | Permanent night shift | 14 (0.5) |  | 11 (1.1) | 3.94 (1.40-11.05) |  | 0 (0.0) | NA ^e^ |
|  | | | Rotating night shift | 1,127 (41.0) |  | 278 (26.8) | 0.95 (0.76-1.19) |  | 22 (26.5) | 1.09 (0.55-2.17) |
|  | | | Irregular night shift | 199 (7.2) |  | 97 (9.3) | 1.56 (1.13-2.14) |  | 9 (10.8) | 2.31 (0.95-5.59) |
| Years of night shift work ^b c^ | | | |  |  |  |  |  |  |  |
|  | | | Daytime work | 1,142 (41.5) |  | 496 (47.7) | 1.00 |  | 39 (47.0) | 1.00 |
|  | | | <5 years | 1,095 (39.8) |  | 253 (24.4) | 0.85 (0.60-1.21) |  | 26 (31.3) | 1.76 (0.72-4.33) |
|  | | | 5-10 years | 286 (10.4) |  | 139 (13.4) | 1.11 (0.75-1.64) |  | 8 (9.6) | 0.36 (0.08-1.71) |
|  | | | ≥10 years | 226 (8.2) |  | 151 (14.5) | 1.25 (0.81-1.91) |  | 10 (12.0) | 1.27 (0.38-4.20) |
|  | | | *p value (test for trend)* |  |  |  | *0.022* |  |  | *0.687* |
|  | | | | Mean±SD |  | Mean±SD | Adjusted OR^*^ (95% CI) |  | Mean±SD | Adjusted OR^*^ (95% CI) |
| Years engaged in night shift work ^c^ | | | | 4.15±5.19 |  | 6.60±6.05 | 1.02 (0.99-1.04) |  | 7.77±7.87 | 1.02 (0.97-1.08) |
| Nights of shifts per week ^d^ | | | | 1.24±0.81 |  | 1.46±1.21 | 1.18 (1.03-1.36) |  | 1.28±0.38 | 1.32 (0.89-1.95) |

^*^ Model 1: In addition to the type of shift work, the variables included in Model 1 are age at interview, sex, marital status, education level, smoking status, drinking habits, consumption of fruit and vegetables, sleep duration, sleep quality, working hours and mental stress; ^a^ Using daytime work as a reference group; ^b^ Using shift work year= 0 as a reference group; ^c^ The variable ‘night shifts per week’ is also included in Model 1; ^d^ The variable “years engaged in night shift work” is also included in Model 1; ^e^ NA: not applicable, as the calculation was not possible due to no cases in this group
