# Supplementary material for: Night shift work exposure profile and obesity: Baseline results from a Chinese night shift worker cohort
Source: PLoS One. 2018 May 15;13(5):e0196989. doi: 10.1371/journal.pone.0196989 (PMC5953447; doi:10.1371/journal.pone.0196989)
Supplement: S7 Table — (DOCX) [file pone.0196989.s007.docx]

S7 Table. Odds ratios (ORs) (removed leisure-time physical activities) and 95% confidence intervals (95% CIs) for the association between the different types of night shift work and abdominal obesity in 3,871 Chinese workers

| Characteristics | | | | Non-abdominal obesity |  | Abdominal obesity | |
| --- | --- | --- | --- | --- | --- | --- | --- |
|  |  |  |  | N (%) |  | N (%) | Adjusted OR^*^ (95% CI) |
| No. of Participants | | | | 2,198 (100.0) |  | 1673 (100.0) | -- |
| Types of shift work ^a^ | | | |  |  |  |  |
|  | Daytime work | | | 854 (38.9) |  | 823 (49.2) | 1.00 |
|  | Night shift work | | | 1344 (61.1) |  | 850 (50.8) | 1.20 (1.01-1.42) |
|  | | Previous night shift work | | 235 (10.7) |  | 202 (12.1) | 1.09 (0.81-1.45) |
|  | | Current night shift work | | 1,109 (50.5) |  | 648 (38.7) | 1.23 (1.03-1.48) |
|  | | | Permanent night shift | 11 (0.5) |  | 14 (0.8) | 3.41 (1.21-9.62) |
|  | | | Rotating night shift | 933 (42.4) |  | 494 (29.5) | 1.17 (0.95-1.43) |
|  | | | Irregular night shift | 165 (7.5) |  | 140 (8.4) | 1.26 (0.94-1.69) |
| Years of night shift work ^b c^ | | | |  |  |  |  |
|  | | | Daytime work | 854 (38.9) |  | 823 (49.2) | 1.00 |
|  | | | <5 years | 964 (43.9) |  | 410 (24.5) | 0.94 (0.68-1.29) |
|  | | | 5-10 years | 224 (10.2) |  | 209 (12.5) | 0.86 (0.59-1.26) |
|  | | | ≥10 years | 156 (7.1) |  | 231 (13.8) | 1.14 (0.73-1.78) |
|  | | | *p value (test for trend)* |  |  |  | *0.095* |
|  | | | | Mean±SD |  | Mean±SD | Adjusted OR^*^ (95% CI) |
| Years engaged in night shift work ^c^ | | | | 3.57±4.72 |  | 6.79±6.23 | 1.04 (1.01-1.06) |
| Nights of shifts per week ^d^ | | | | 1.25±0.80 |  | 1.38±1.09 | 1.09 (0.95-1.24) |

Non-abdominal obesity, waist circumference ≤85 cm for men or ≤80 cm for women; Abdominal obesity, waist circumference >85 cm for men or >80 cm for women;

^*^ Model 1: In addition to the type of shift work, the variables included in Model 1 were age at interview, sex, marital status, education level, smoking status, drinking habits, consumption of fruit and vegetables, sleep duration, sleep quality, working hours and mental stress; ^a^ Using daytime work as a reference group; ^b^ Using shift work year= 0 as a reference group; ^c^ The variable ‘night shifts per week’ was also included in Model 1; ^d^ The variable “years engaged in night shift work” was also included in Model 1.
